# Insights into AIE materials: A focus on biomedical applications of fluorescence

**DOI:** 10.3389/fchem.2022.985578

**Published:** 2022-09-15

**Authors:** Junchi Ma, Yanru Gu, Depeng Ma, Weizhao Lu, Jianfeng Qiu

**Affiliations:** ^1^ Translational Medicine Research Centre, The First Affiliated Hospital of Shandong First Medical University and Shandong Provincial Qianfoshan Hospital, Jinan, China; ^2^ College of Radiology, Shandong First Medical University and Shandong Academy of Medical Sciences, Jinan, China

**Keywords:** AIE (aggregation-induced emission), fluorescence, biotesting, bioimaging, material

## Abstract

Aggregation-induced emission (AIE) molecules have garnered considerable interest since its first appearance in 2001. Recent studies on AIE materials in biological and medical areas have demonstrated that they show their promise as biomaterials for bioimaging and other biomedical applications. Benefiting from significant advantages of their high sensitivity, excellent photostability, and good biocompatibility, AIE-based materials provide dramatically improved analytical capacities for *in vivo* detection and demonstration of vital biological processes. Herein, we introduce the development history of AIE molecules and recent progress in areas of biotesting and bioimaging. Additionally, this review also offers an outlook for the potential applications of versatile AIE materials for tracing and treating pathological tissues, including overcoming challenges and feasible solutions.

## Graphical Abstract

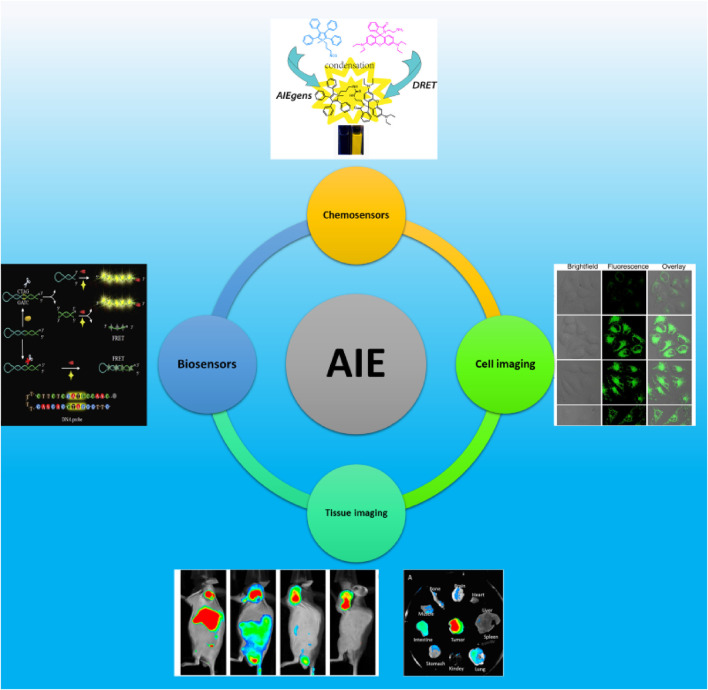

## Highlights

- The history of the development of AIE molecules is narrated and discussed.
- Different kinds of AIE structures are summarized.
- The properties of sensing and imaging are exhibited as separate listings.
- Challenges in designing and applications are considered and outlooked.

## Introduction

Over the past several decades, the discovery and design of small organic molecules capable of fluorescence imaging have become a rapidly expanding area of research (Maity et al., 2015; Jia and Wu, 2020a; Zhang et al., 2020a; Younis et al., 2020; Zhu et al., 2020), particularly owing to their possible practical applications in environmental monitoring, tissue engineering (Dong et al., 2020; Song et al., 2020; Zeng et al., 2020), and medical examination and treatments (Hu et al., 2020a; Jia and Wu, 2020b; Yu et al., 2020). As a powerful tool for tracing, demonstrating, and analyzing biomolecules such as esters (Cai et al., 2020), carbohydrates (Qu et al., 2020), peptides (Gao et al., 2020a), enzymes (Dong et al., 2020a; Zhao et al., 2020a), and nucleotides (Zhang et al., 2020b), fluorescent biosensors provide deep insight into the complicated *in vivo* chemical, biological, and physiological processes (Wang et al., 2019) to explore authentic pathogenesis and precise diagnosis. Under these circumstances, the research and development of fluorescent biosensors with superior performance are significant.

**FIGURE 1:**
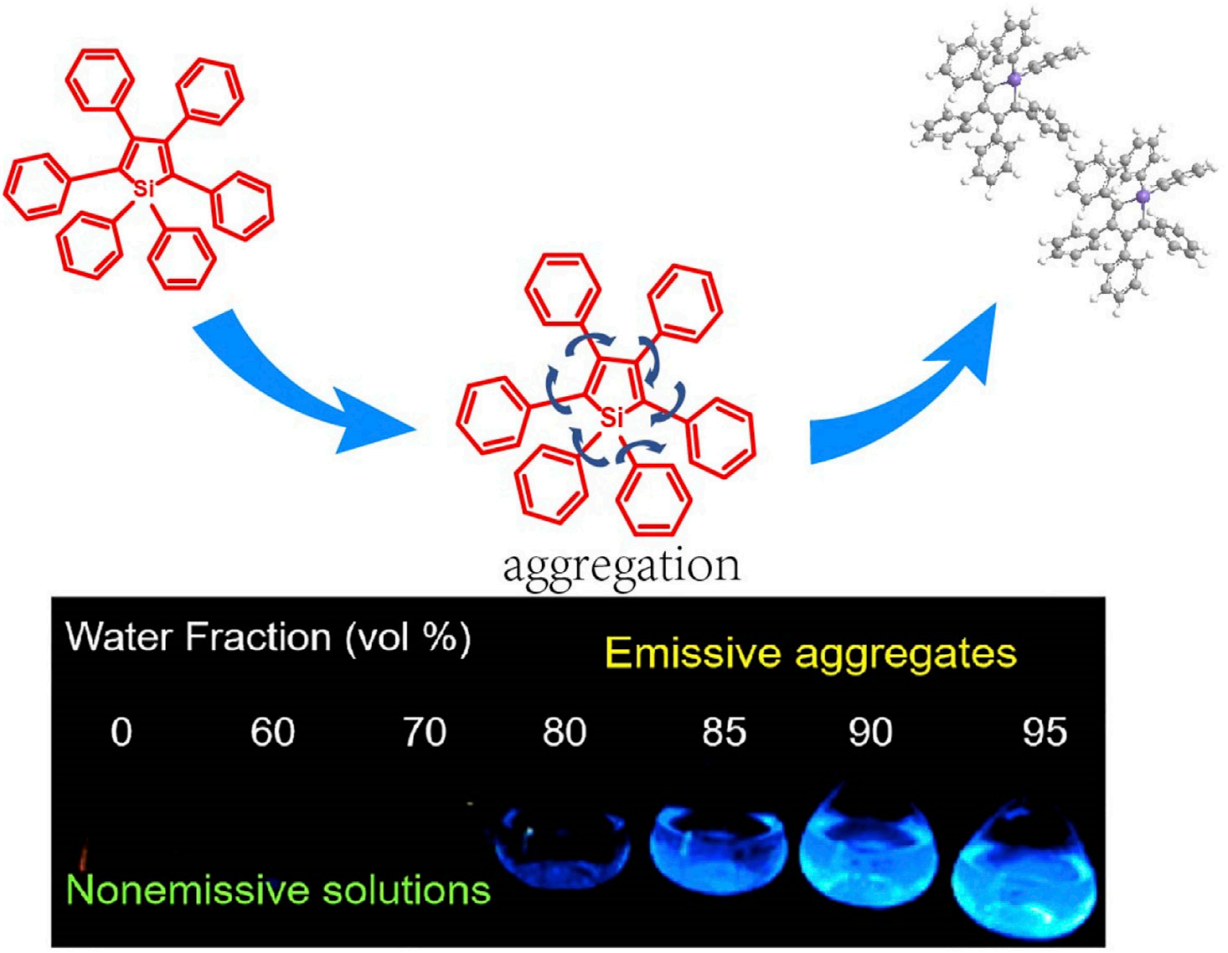
Aggregation-induced emission effect: fluorescence photographs of silole molecules in THF/water mixtures under UV light (Dong et al., 2020a).

To achieve this, suitable fluorescence molecules and materials for *in vivo* tracing and demonstration are key. The optical properties of most organic dyes usually depend on their large conjugate structure, which may be extremely weak owing to their poor water solubility as well as the fluorescence quenching in the aggregate state, also known as the vicious aggregation-caused quenching (ACQ) effect. This phenomenon could be explained as tedious conjugation with aromatic rings accumulated via strong π–π interactions. This ACQ effect of conventional organic fluorescence molecules makes the design of desirable fluorescent structures challenging. In this case, the development of molecules that can overcome the disadvantages of the ACQ effect is extremely urgent. Fortunately, fluorophores with aggregation-induced emission (AIE) property provide a clear path to achieve this since Tang (Luo et al., 2001) first discovered this dramatic phenomenon in 2001. As is shown in Figure 1, AIE fluorophores exhibit bright fluorescence in the aggregated state but very weak fluorescence in good solvent, making them ideal “turn-on” fluorescent probes for bioanalysis (Chen et al., 2020a; Huang et al., 2020a; Li et al., 2020a; Yang et al., 2020a; Zhang et al., 2020b; Korzec et al., 2020). With sufficient special and unique optical properties, including their simple molecular structure (Pratihar et al., 2020), high SNR (signal-to-noise ratio) (Xu et al., 2020a), high luminescence efficiency (Ma et al., 2020a; Fei et al., 2020; Leng et al., 2020), excellent photostability (Jiang et al., 2020a; Mondal et al., 2020a; Wei et al., 2020), and biocompatibility (Xu et al., 2020b; Zhu et al., 2020), AIE fluorescent probes have been widely used as fluorescent components of molecular probes and nanoprobes for biomedical applications in clinical examination and therapy as well as in biological sensing (Gao et al., 2020b; Zhao et al., 2020b; Xu et al., 2020c; Su et al., 2020) and *in vivo* theranostics.

Herein, we aim to analyze recent advances in AIE molecules and its applications in biotesting and bioimaging, as well as their impact on in medical theranostics. We studied categories of AIE molecules to comprehend the relationship between the luminophore structure and optical properties, the biotesting applications of AIE sensors with target quantitative analysis, and the bioimaging applications of visual demonstration. Finally, we share our outlook about the use of AIE molecules and materials for physiological application.

## Categories of AIE materials

**FIGURE 2:**
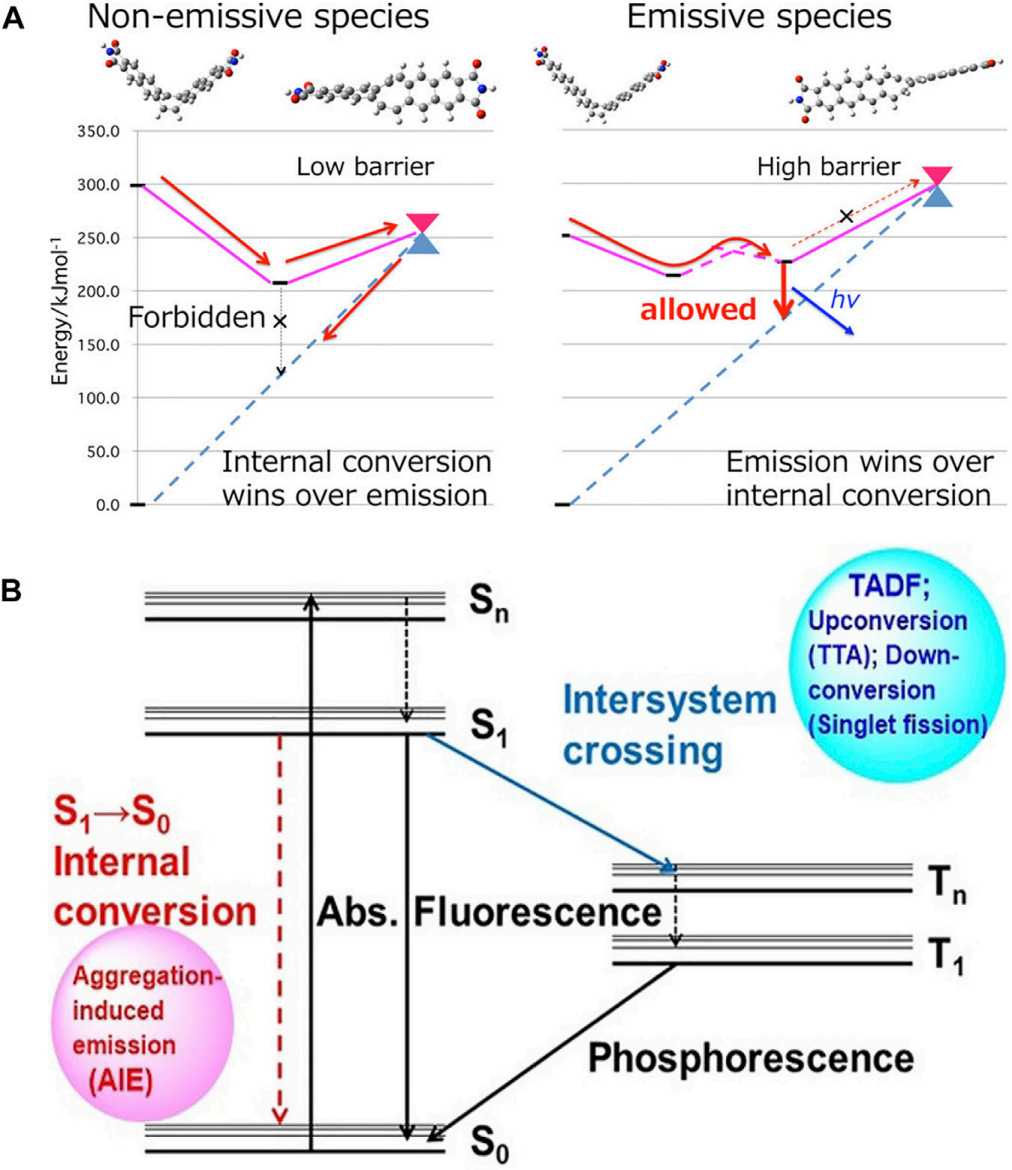
**(A)** Schematic illustration of the conical intersection accessibility in dilute solution (left) and aggregates (right) (Suzuki et al., 2015). **(B)** Jablonski diagram of an atypical organic fluorophore (Suzuki et al., 2020).

The restriction of intramolecular motion (RIM) is the basis of the AIE effect (Mei et al., 2014), which includes the restriction of intramolecular rotation (RIR) and the restriction of intramolecular vibration (RIV). Independent quantum chemical investigations have been conducted aiming for thorough understanding of the relationship between intramolecular motion and AIE properties. Suzuki et al. (2015) explored quenching pathways of three multiluminescent molecules and predicted a largely distorted structure near the minimum energy conical intersection (MECI). The MECI state is easy to achieve in solution but difficult to achieve in solid state and impossible to achieve in crystalline state (Figure 2A). As demonstrated in Figure 2B, the fluorescence quenching of AIEgens in solution must occur through an internal conversion process (S_1_→S_0_), whose efficiency is directly affected by the restrictions of intramolecular motions (Suzuki et al., 2020).

**FIGURE 3:**
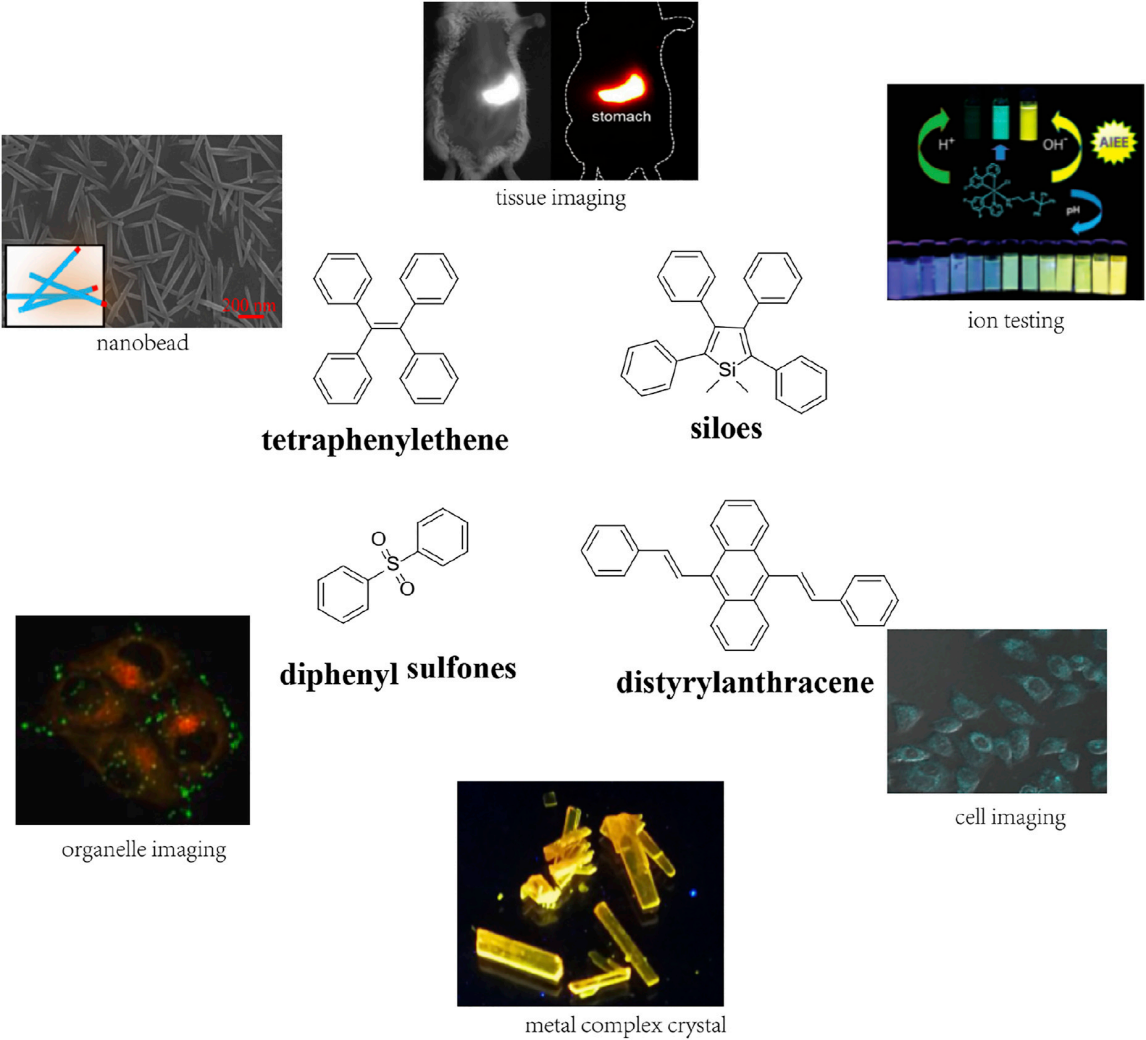
The most prominent AIE backbone structures commonly developed in recent research.

Since Tang first reported hexaphenylsilole (HPS) as the first AIEgen, numerous fluorogens with AIE properties such as tetraphenylethene (Pradhan et al., 2015; Zhao et al., 2020a; Huang et al., 2020b; Jiang et al., 2020b; Zhao L. et al., 2020b; Zhang et al., 2020c; Zhan et al., 2020), siloes (Zhang et al., 2020a), diphenyl sulfones (Zheng et al., 2020), and distyrylanthracene derivatives (Diao et al., 2020; Zhang et al., 2020e) have been studied for analysis and exhibition (Figure 3). Mechanically, most have a non-coplanar structure to reduce the energy barrier. Owing to their small size and low steric hindrance, AIEgens can be modified by cross-linking, esterification, grafting, and molecular and compositional modification to improve their capabilities.

The pattern of manifestation is also important for AIEgens to improve their performance. Synthetic AIE materials such as metal nanoparticles (Munirathnappa et al., 2020; Wu et al., 2020), nonconventional polymers (Dong et al., 2020b; Ma H. et al., 2020b; Yang et al., 2020b; Huang et al., 2020c; Zhang et al., 2020f; Xue et al., 2020), and nanodots (Dong et al., 2020a; Crucho et al., 2020) are generally free of aromatic building blocks and conjugated structures. These synthetic AIEgens generally display concentration-enhanced emission and aggregation-induced emission (AIE) characteristics. Given the presence of these systems, the clustering-triggered emission (CTE) mechanism could help explain the photophysical processes of these synthetic AIEgens (Hu et al., 2020b; Lu et al., 2020).

## Application of biotesting

### Chemosensor

Compared with the other detection methods, AIE-based fluorescent chemosensors show promise in the detection of ions (Liu et al., 2020; Wang Q. et al., 2020; Li et al., 2020; Wang et al., 2020; Guan et al., 2020; Zhou et al., 2020) and organic molecules (Yan et al., 2020; Yang et al., 2020; Zhao et al., 2020; Xu et al., 2020b; Feng et al., 2020; Rajalakshmi and Palanisami, 2020). According to the excited-state intramolecular proton-transfer (ESIPT) mechanism, the fluorescent properties of the fluorophore skeleton are highly dependent on the solvent environment due to the variation of the intramolecular hydrogen bond.

### pH sensor

As one of the most important parameters in chemical, physiological, and biological processes, pH plays an indispensable role in the qualitative and quantitative analysis chemistry. In particular, subtle changes in intracellular pH associated with various cellular processes provide ample information on the studied organisms.

For the past few years, pH tracer based on fluorescence responses has been designed and developed. The commonly used fluorescent structures cover the emission region from visible to near-infrared (NIR), such as tetraphenylethene (TPE) (Chen et al., 2019), coumarin (Meimetis et al., 2014), naphthalimide (Ramasamy and Thambusamy, 2017), rhodamine (Mao et al., 2019), boron dipyrromethene (BODIPY) (Li et al., 2016), and cyanine (Cy) (Hou et al., 2017). To take advantage of the varied classifications of the fluorophores, it is easy to find out the common functional mechanism of these probes: structure transformation attributed to protonation and deprotonation in fluorescein, which makes them strong pH probe candidates.

**FIGURE 4:**
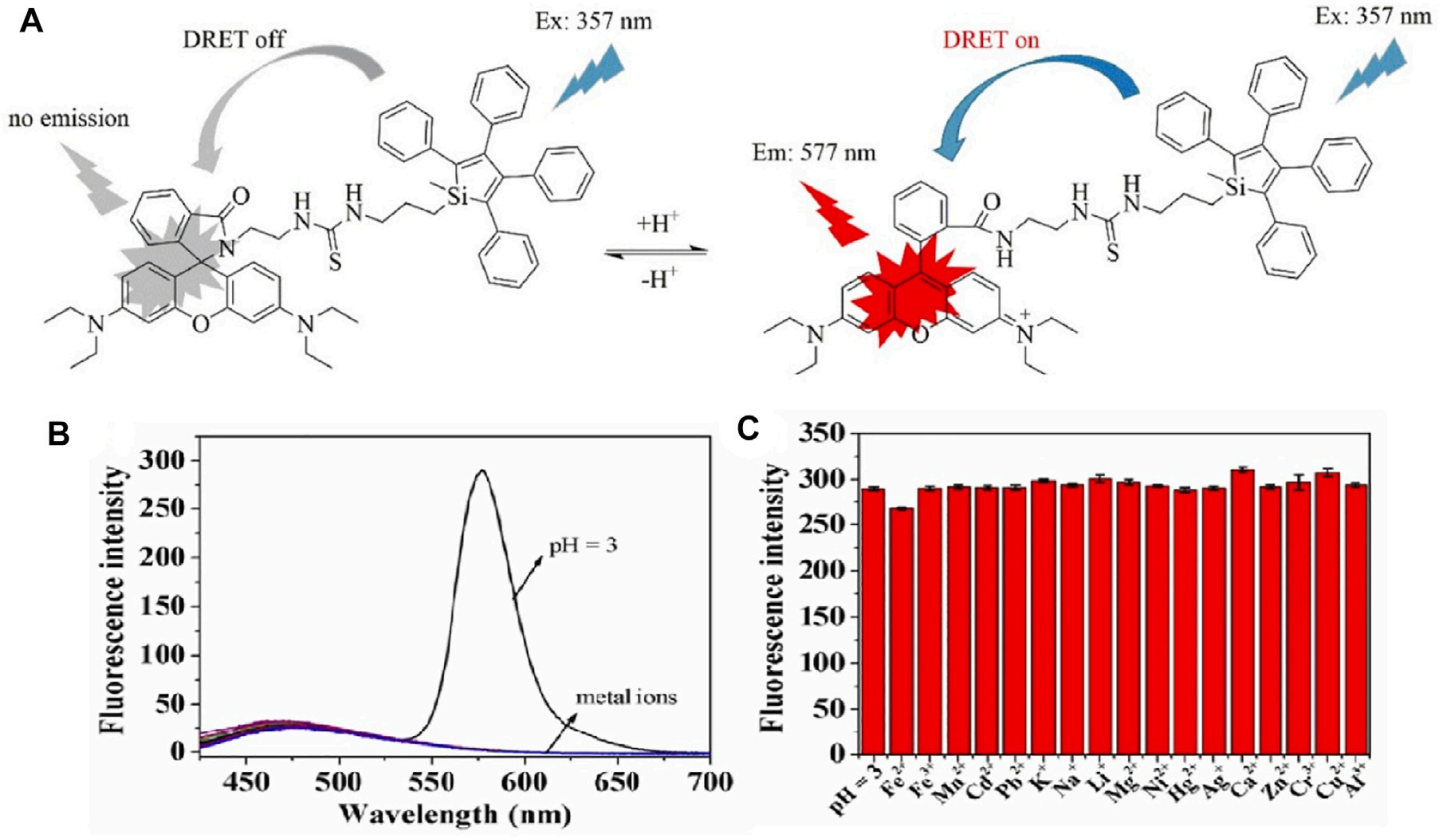
**(A)** Fluorescence properties of silole derivatives in mixed methanol and citrate buffer with adjustment of pH; **(B)** fluorescence responses of SRH to pH value and metal ions under an excitation of 357 nm. **(C)** Fluorescence intensity of SRH in mixed methanol and citrate buffer in the presence of marked metal ions.

To date, given the diversity of designing fluorescence molecules, the majority of the existing AIE pH probes were developed through the molecular engineering of electron donors, acceptors, spacers, and D–A conjugated groups (Liu S. et al., 2020). A silacyclopentadiene derivative was the first discovered AIEgens. This was combined with the rhodamine B structure to create an energy donor and acceptor (Wang X. et al., 2020a), which eliminate fluorescence quenching from the non-emissive character of the silole group in an aqueous solution. Complexation with hydrion results in the dredging elutriate test (DRET) procedure and leads to dramatic fluorescence enhancement. As shown in Figure 4, this mixed-mode probe responds to pH from 2.75 to 5, with a red shift of 220 nm**.**

**FIGURE 5:**
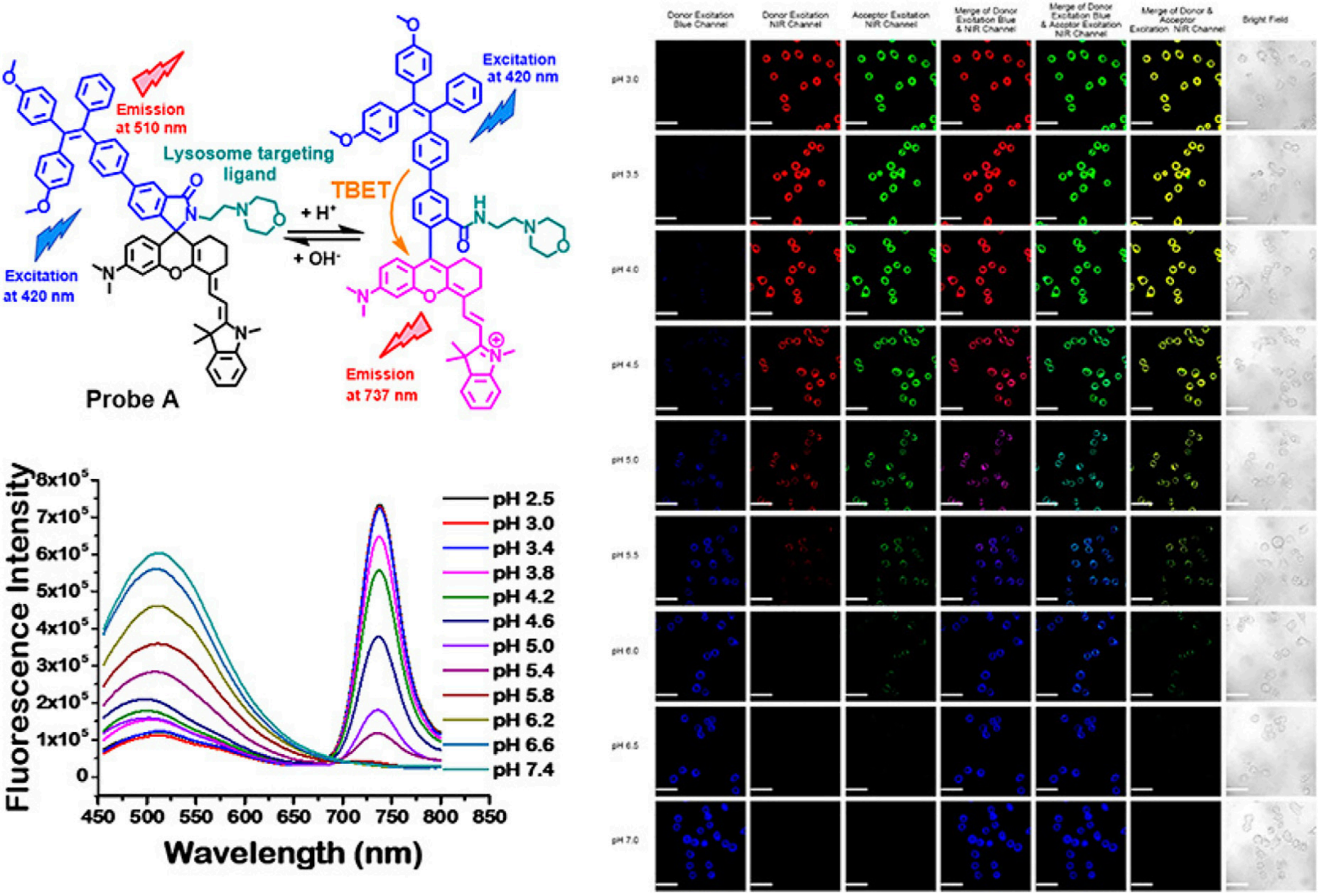
Chemical structure responses of ratiometric near-infrared fluorescent probes to pH changes; fluorescence spectra of 5 µM probe A in 10 mM citrate buffers with different pH values, containing 30% ethanol under an excitation of 420 nm; fluorescence images of HeLa cells incubated with 15 µM fluorescent probe A (Wang et al., 2018).

Among numerous studies, intracellular pH sensing is a characteristic work in which more attention has been paid to biomedical application. In Figure 5, Liu (Wang et al., 2018) designed a ratiometric probe with a TPE donor and a NIR hemicyanine acceptor for sensitive detection of pH changes in live cells. This strategy can be used to develop a variety of novel proportional fluorescent probes to accurately detect different analytes in the chemical reaction by introducing appropriate sensing ligands into the hemicyandiamide group to form the spironolactone switch.

### Ion sensor

Being the main form of existence of metals in environmental and living systems, ions can be further divided into cations and anions and possess important implications for all physiological processes. To figure out the quantification and thermal decomposition of ions, chemosensors with AIEgens for ion detection have been studied extensively (Yan L. et al., 2020; Zhai et al., 2020). The commonly used method is the probe that binds with metal ions through noncovalent interactions (mainly metal–ligand coordination), and this recognition process is in a reversible manner. In contrast, another strategy involves an irreversible chemical reaction as induced by a target metal ion that acts as either a reactant or a catalyst, leading to forming a new product after the reaction. Thus, the unique reaction-based indicators are also described as chemodosimeters.

**FIGURE 6:**
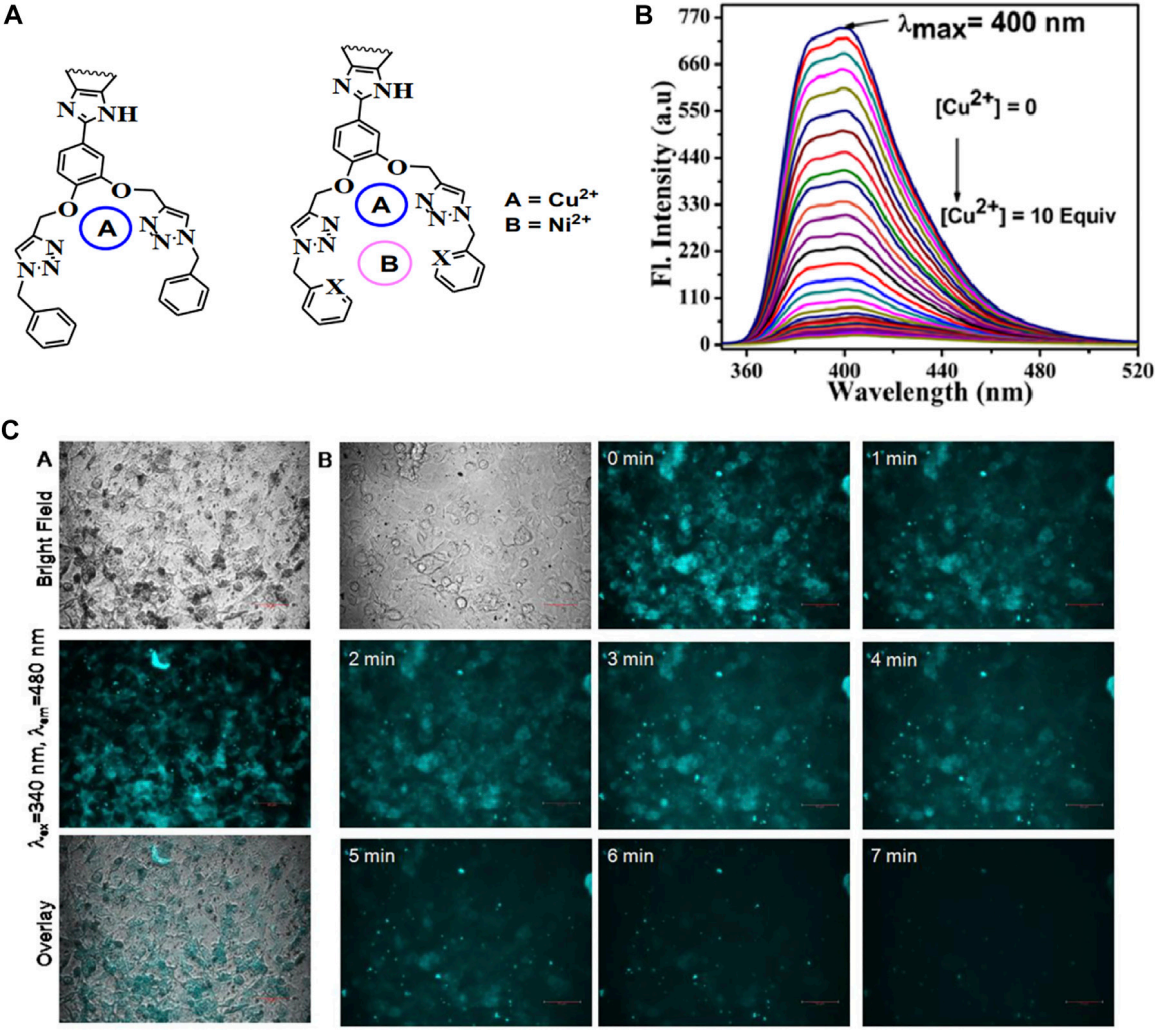
**(A)** Dual-cavities and probable binding mode of the sensors; **(B)** emission spectra of probe P3 with the incremental amount of Cu^2+^ in ACN; **(C)** fluorescent bio-images of C6 (rat glial cell) incubated with P3 and cell incubated with P3 along with Cu^2+^ with different time intervals (Mondal S. et al., 2020).

Nitrogen heterocycles with lone pairs of electrons often exhibit aggregation-induced emissive properties. Based on this, Surajit (Mondal S. et al., 2020) designed and prepared triazole-based molecules with outstanding AIE effects (Figure 6). Formed by reacting ether-linked-triazole and pyridine with copper ions intramolecularly, the probe could recognize Cu^2+^ with a low limit of detection (LOD) in the nM range, demonstrating its high sensitivity, and can be explained by the mechanism of the photoinduced electron transfer (PET) process. Cytotoxicity tests confirmed the potential of the system as a novel cell imaging technique.

**FIGURE 7:**
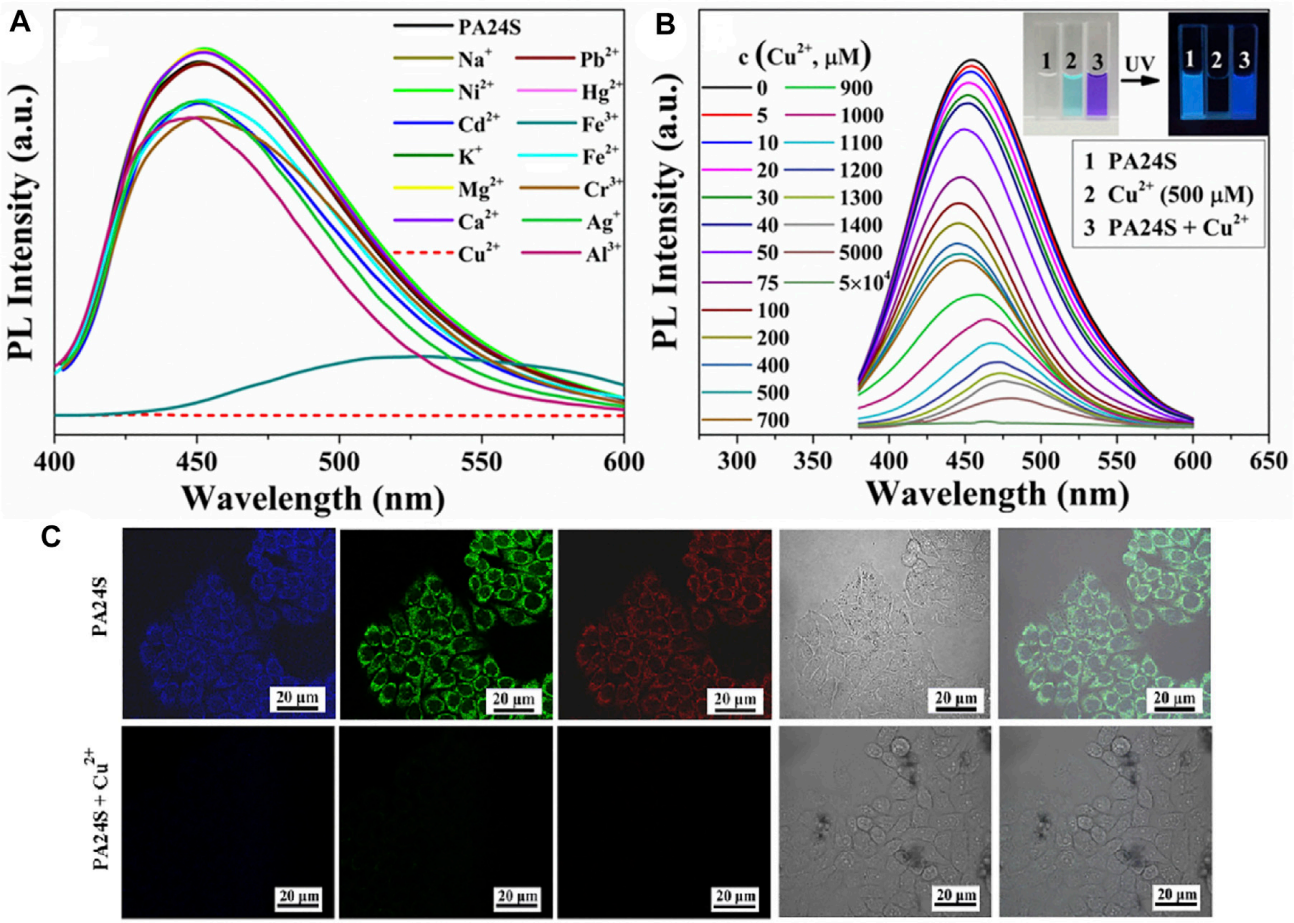
**(A)** Fluorescence emission spectra (λ_Ex_ = 370 nm) of PA24S solution in the presence of different metal ions; **(B)** those of PA24S solution as a function of Cu^2+^ concentration; **(C)** images of CFM of HeLa cells cultured for 30 min in the presence of PA24S and Cu^2+^ under 405 nm, 488 nm, 561 nm, bright field, and their overlay (Wang Q. et al., 2020).

Without typical luminophores in its structure, the emission behavior of the ethylene succinamide salt PA24S regarding numerous physicochemical variable parameters as its acid–base properties, temperature, concentration, and anti-interference ability has been discussed by Qinghui Wang et al. (2020). As shown in Figure 7, it was found that the fluorescence emission functioned through aggregation and molecular clusters, which was verified by its spectroscopic properties, micromorphology, and structural characterization by NMR. The probe showed significant sensitivity to Cu^2+^ in an aqueous solution with effective fluorescence quenching, indicating that PA24S exhibited superior for Cu^2+^ detection ability and visual monitoring.

**FIGURE 8:**
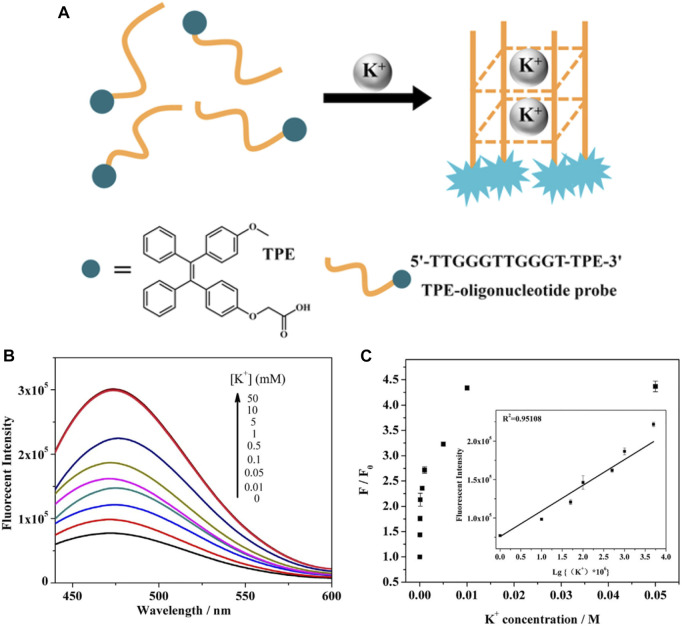
**(A)** Schematic illustration for TPE-oligonucleotide K^+^ probe; **(B)** fluorescence spectra for different concentrations of K^+^ based on the AIE effect of TPE-oligonucleotide probe. **(C)** Plot of fluorescence enhancement (F/F_0_) at 475 nm as a function of K^+^ concentration (Lu et al., 2017).

Associated with the regulation of biochemical signal transduction in the nervous system, potassium plays critical roles in biological processes including muscle contraction, cardiac motion, nerve conduction, and urinary functions. Fluorescent probes with crown ethers and other structures have been developed and attracted significant attention. Lu (Lu et al., 2017) designed a novel G-quadruplex structure-based K^+^ probe (Figure 8). Benefit from the TPE AIEgen, the probe exhibits high sensitivity with extended photostability, which facilitates the prolonged fluorescence observations of K^+^ in living cells.

**FIGURE 9:**
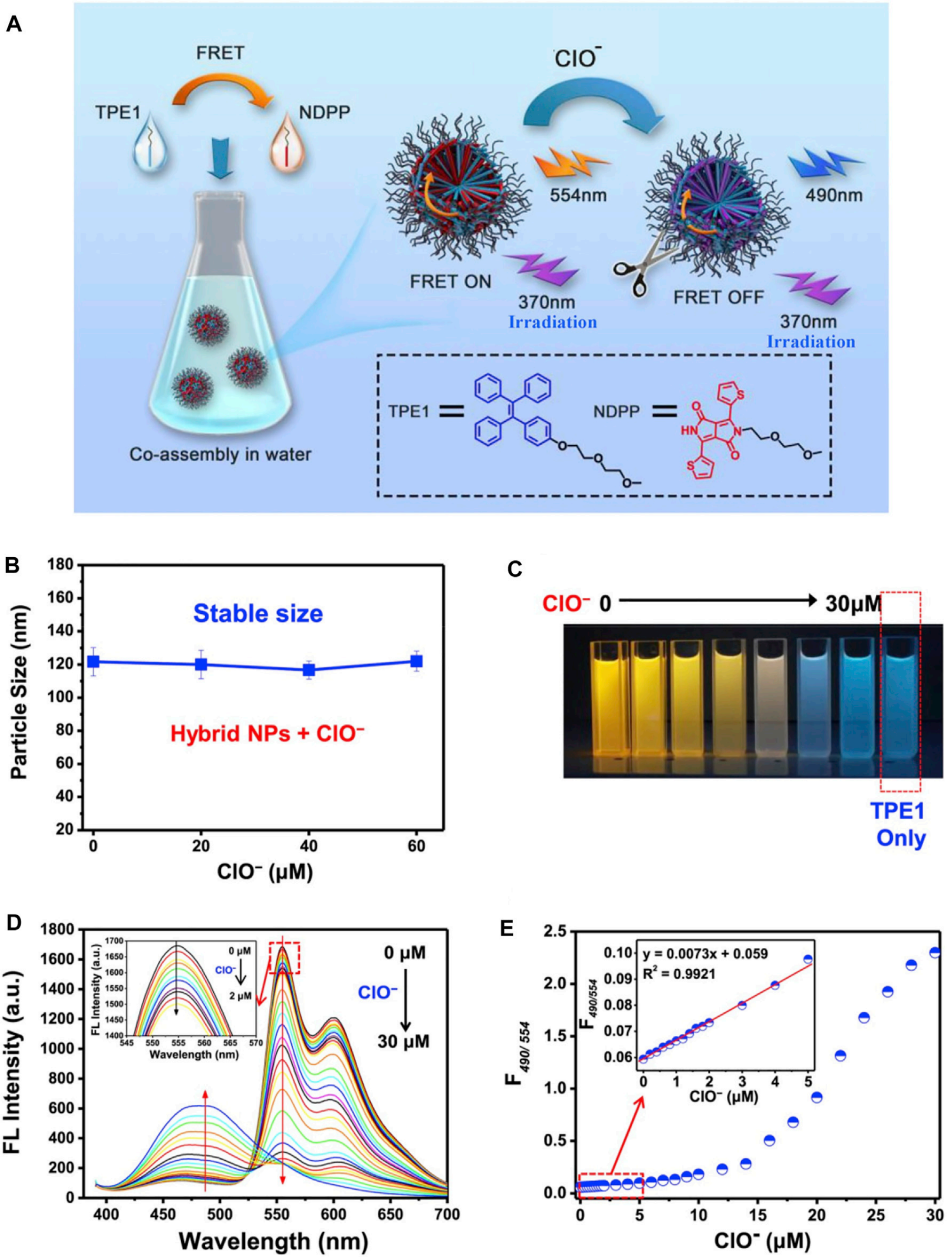
**(A)** Construction of a micellar FRET nanoprobe composed of a TPE/NDPP pair and the effect of ClO-; **(B)** changes in the DLS size of TPE1-NDPP NPs upon the addition of increasing amounts of ClO^−^ in water; **(C)** photons of a solution with TPE1-NDPP NPs and different amounts of ClO^−^ (0, 4, 8, 16, 20, 25, and 30 μM) under a UV lamp; **(D)** changes in the fluorescence spectra of the TPE1-NDPP NPs upon the addition of increasing amounts of ClO^−^ (1–30 μM) in water. Inset: the addition of a low concentration of ClO^−^ (0, 0.2, 0.4, 0.6, 0.8, 1.0, 1.2, 1.4, 1.6, 1.8, 2.0 μM); and **(E)** fluorescence intensity ratio (F490/554) changes of TPE1-NDPP NPs upon exposure to ClO^−^ (Nie et al., 2020a).

Nanoparticles with AIEgens hold tremendous potential as a tracer material, for they can penetrate biotic tissues with no damage. AIE-based fluorescence resonance energy transfer (FRET) nanoparticles (Nie et al., 2020a) were developed to recognize ClO^−^ in living cells (Figure 9). Different from common FRET-based nanosensors, the NPs were prepared via facile co-assembly strategy in water combining amphiphilic tetraphenylethene with thienyl-diketopyrrolopyrrole. The fluorescent imaging of the probe with ClO^−^ demonstrates how NPs can effectively pass through the cell membrane and safely recognize ClO^−^ in living cells.

### Molecular sensor

**FIGURE 10:**
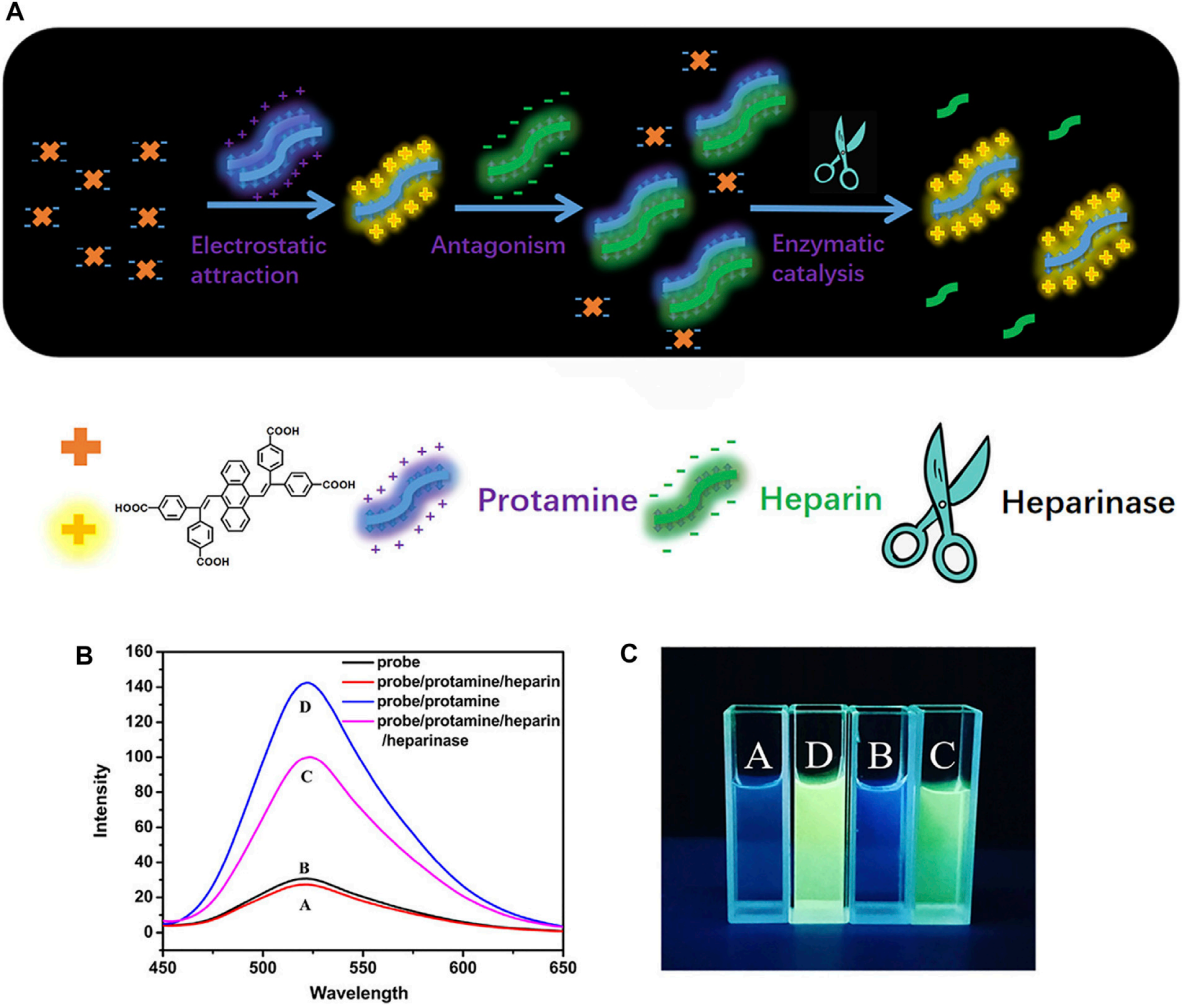
**(A)** Self-assembly of the copolymer (HSP-TPE) containing fluorescent TPE probe and sensing behavior of HSP-TPE aggregates toward picric acid (PA); **(B)** fluorescence spectra of DSA-4COOH, DSA-4COOH/PRO/HEP complex, DSA-4COOH/PRO/HEP/heparinase complex, and DSA-4COOH/PRO complex; and **(C)** photograph of digital photos of DSA-4COOH with protamine, heparin, and heparinase under irradiation at 365 nm.

In consideration of the different properties of objects including charge, hydrophobicity, bioactivity, chemical activity, etc., numerous sensors have been designed and developed for monitoring small molecules by rationally modulating recognition units and luminescent units (Yao et al., 2011). A distyrylanthracene-based molecule was developed with four carboxyl groups to improve the combining capacity toward amino acids (Jiang R. et al., 2020a). As shown in Figure 10, with structure similar to tetraphenylethene (TPE), this convenient and sensitive fluorescent probe with AIE character can distinguish protamine, heparin, and heparinase. The detecting mechanism was found to be aggregation caused by electrostatic attraction and enhanced the fluorescence of the system.

**FIGURE 11:**
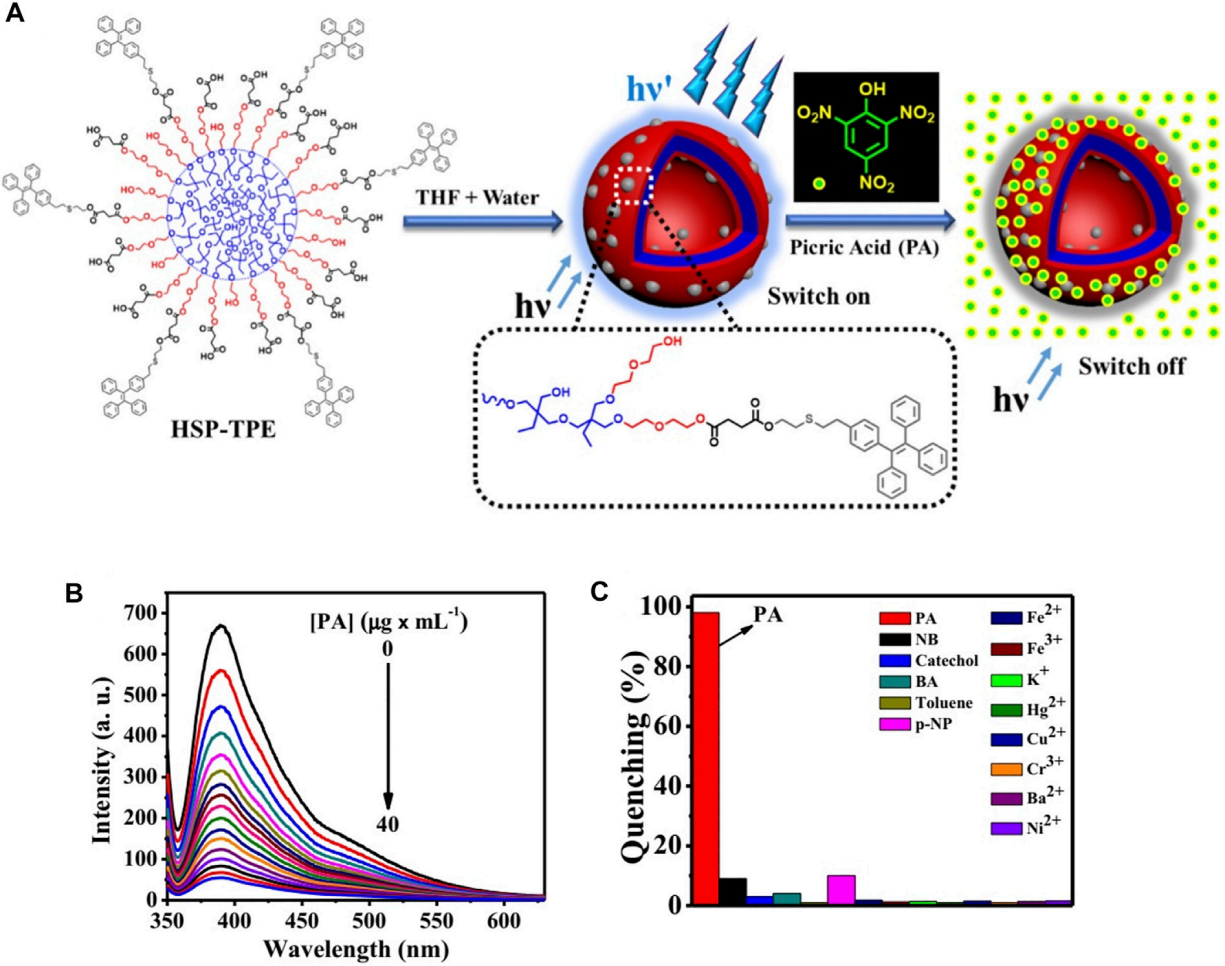
**(A)** Illustration of self-assembly of the copolymer (HSP-TPE) containing fluorescent TPE probe and sensing behavior of HSP-TPE aggregates toward picric acid (PA); **(B)** fluorescent spectra of **(A)** HSP-TPE in THF/water (3:7 v/v) mixture with varied amount of picric acid (PA) at an excitation wavelength of λ = 333 nm; **(C)** extent of fluorescence quenching efficiency of HSP-TPE by adding 10 equiv of picric acid (PA) and other molecules (Nabeel et al., 2020).

Furthermore, the detection of other molecules, such as pesticide, toxin, and explosives (TNT, TNP, etc.), also has great implications for personal and social safety. TPE-grafted hyperbranched copolymers (Nabeel et al., 2020) were used to form a homogeneous solution in THF for the sensitive detection of picric acid (PA) (Figure 11). The aggregates showed great fluorescence enhancement in the THF/water mixture solution upon addition of TPE units to the polymers. This granted them high sensitivity to PA, with detection limits as low as 20 PPB. To a certain extent, it can create safer environments and reduce risk factors.

### Biosensors

Detection of the biomolecules in the body fluid appears to be essential for disease diagnosis, monitoring, and management (Sargazi et al., 2022). Owing to their simple structure, low background noise, excellent photostability, and admirable biocompatibility, AIE molecules are beginning to establish themselves as significant tools in the areas of life science for biosensor (Zhang L. et al., 2020b; He et al., 2020) and comprehending bioprocesses (Wang et al., 2015; Gu et al., 2017; Gao F. et al., 2020). To elucidate the biological functions of fluorescent probes or drugs (Wang Z. et al., 2020), a comprehensive understanding of their intracellular division is of vital importance (Dong Y. et al., 2020; Tang et al., 2020). The most common pattern for detecting biological macromolecules is functionalizing with small hydrophilic groups such as amine groups, sulfonic acid groups, or hydrophilic peptide chains to greatly improve the water solubility of AIE molecules and then utilizing the electrostatic interaction, hydrophobic interaction, and receptor-assisted interactions between the water-soluble AIE molecules and biological macromolecules to turn on/off the fluorescence and achieve the detection (Xu et al., 2012).

**FIGURE 12:**
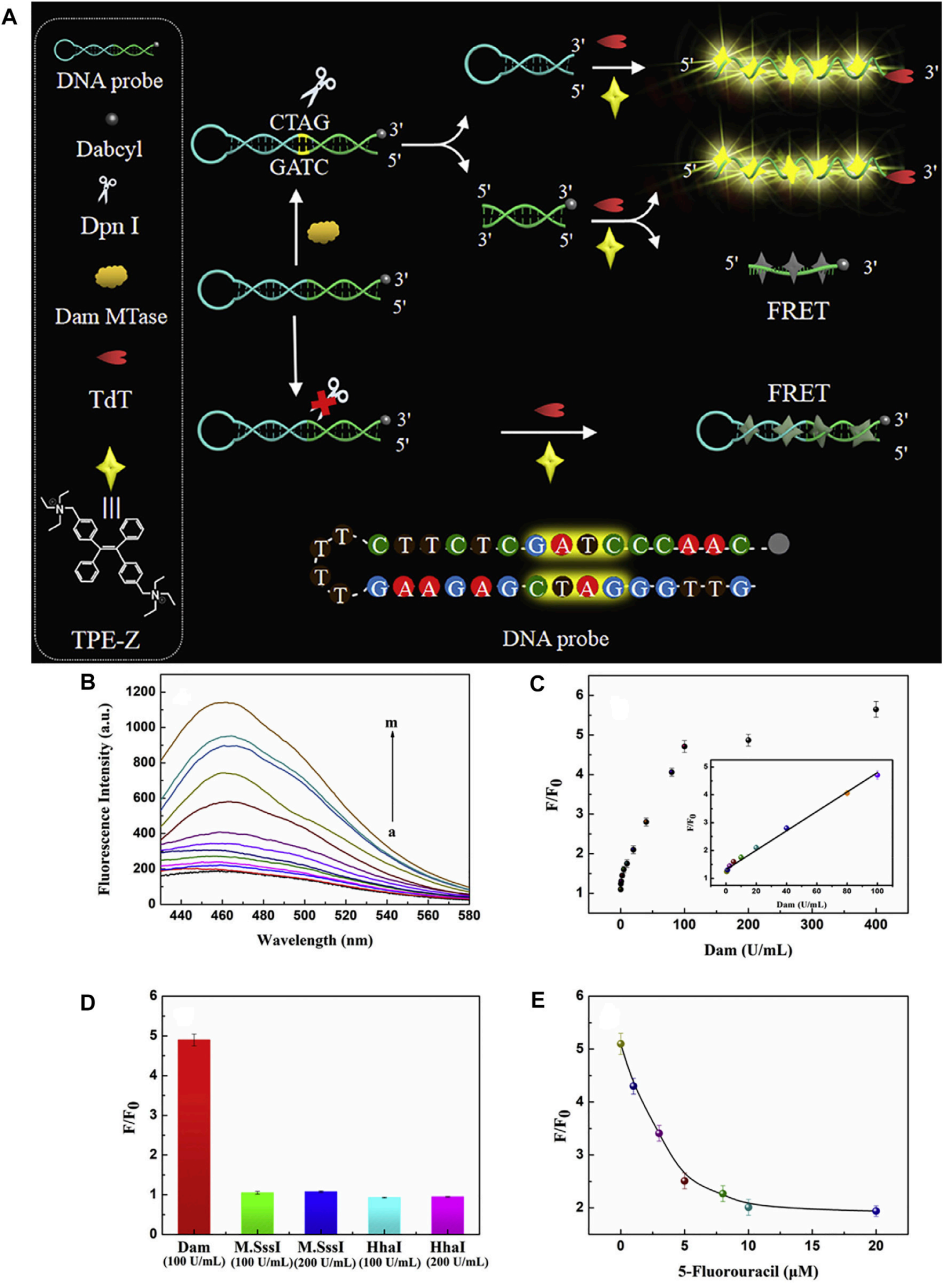
**(A)** Illustration of Dam MTase activity assay based on aggregation-induced emission and template-free DNA polymerization. **(B)** Fluorescence spectra of TPE-Z in response to Dam MTase in different concentrations (0, 0.1, 0.5, 1, 2.5, 5, 10, 20, 40, 80, 100, 200, 400 U/mL). **(C)** Corresponding fluorescence ratio at 462 nm. The inset represents the linear relationship between the signal and Dam MTase concentrations from 0.5 U/mL to 100 U/mL. **(D)** Selectivity study. **(E)** Inhibition assay for Dam MTase with different concentrations of 5-fluorouracil. The error bars were based on three repetitive experiments performed (Niu et al., 2020).

As discussed above, AIE fluorophores (triphenylethene and tetraphenylethene) can be conjugated with biomolecules by condensation or click-reaction, in order to enhance their biocompatibility and bioactivity. Niu et al. (2020) chose quaternized tetraphenylethene salt as the AIE fluorescent module probe, which binds to single-stranded DNA by the electrostatic interaction. As shown in Figure 12, in the presence of DNA MTase, the methylation reaction-initiated DNA polymerization occurs with terminal deoxynucleotidyl transferase (TdT), which activated the fluorescence intensity through AIE. The assay was also effective for the detection of DNA MTase activity in human serum, demonstrating the inhibitory effect of 5-fluorouracil on Dam MTase.

**FIGURE 13:**
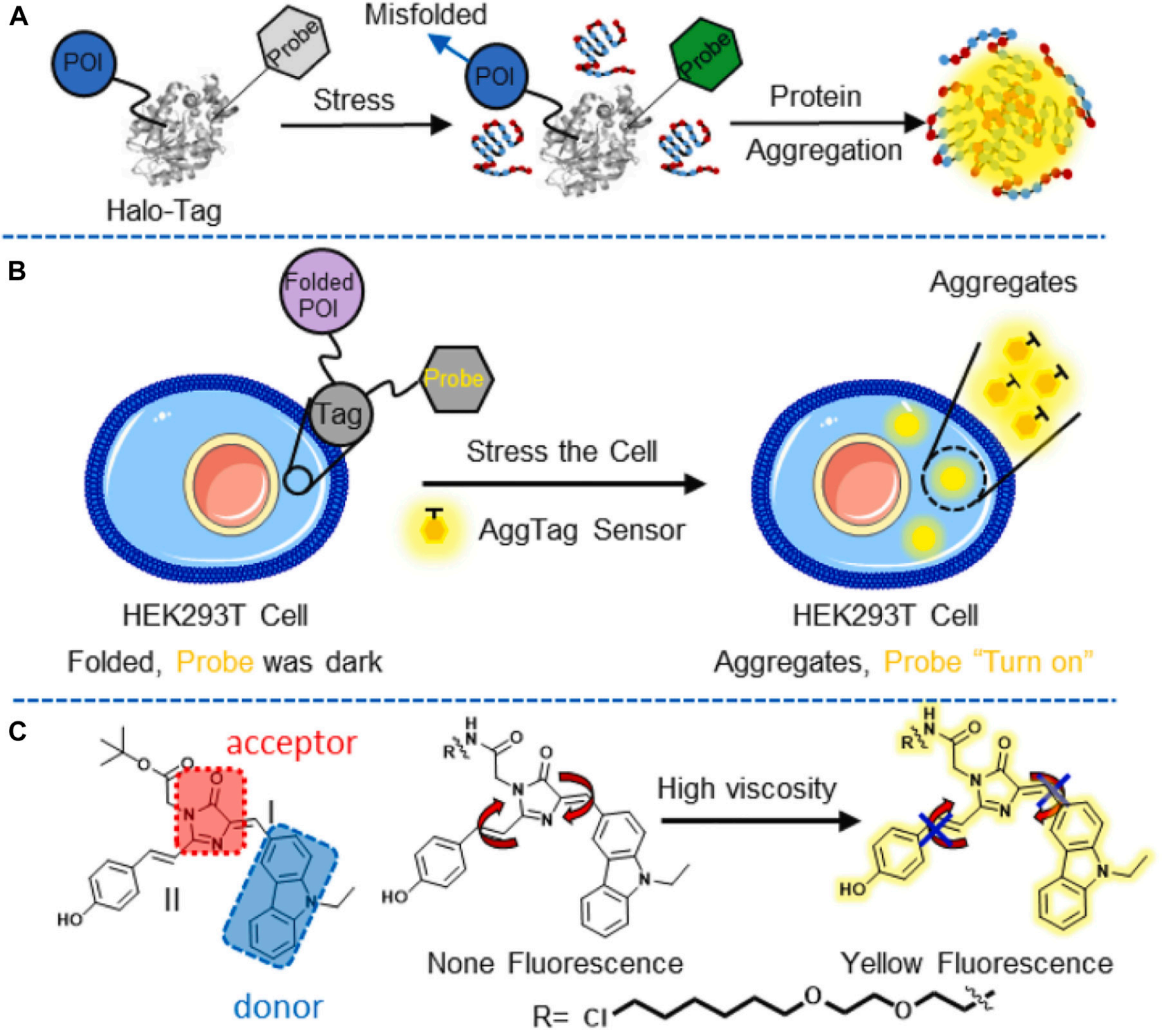
**(A)** Illustration of the Agg-Tag method; POI: protein-of-interest, Probe: Agg-Fluor probes; **(B)** schematic demonstration of the probe detection of aggregated proteins in HEK293T cells; and **(C)** fluorescence of the probe is turned on by rotation restriction at I and II region in highly viscosity microenvironment (Liu et al., 2022).

Since proteins are the key components of the organelle, the quantitative study thereof is important for understanding the basic principle of cellular systems and the related diseases (Olzscha et al., 2011; Burslem and Crews, 2017). It is essential to realize the progress of protein aggregation in biological samples before investigating effective therapies. Liu et al. (2022) developed a series of AIE probes based on 4-hydroxybenzylidene-imidazolinone (HBI) (Figure 13). The varying viscosity sensitivities of AIEgens were explored systematically to visualize protein aggregation in live cells, and other biological processes related to local viscosity changes were also investigated.

## Application of bioimaging

To garner powerful tools for cancer diagnosis and medicinal therapy, fluorescent tracers and dyes have been generally fabricated by encapsulating AIEgens into lipid or BSA shells through a nanoprecipitation route, which allows further surface functionalization with specific recognition units (Wang X. et al., 2020b). The inherent ACQ effect of conventional organic luminophores has been a thorny problem to solve, while the AIE molecule offers more possibility for tracing and mapping special tissues. Because of their low cytotoxicity and high quantum yield, AIE tracers can achieve bioimaging in living systems (Nie et al., 2020b; Zhao W. et al., 2020; Wang X. et al., 2020c). In this case, the development of AIE tracers that enable the *in vivo* monitoring and long-term tracing biological processes with high resolution and sensitivity is of critical importance in both fundamental biological science and practical clinical applications.

### Cell imaging

**FIGURE 14:**
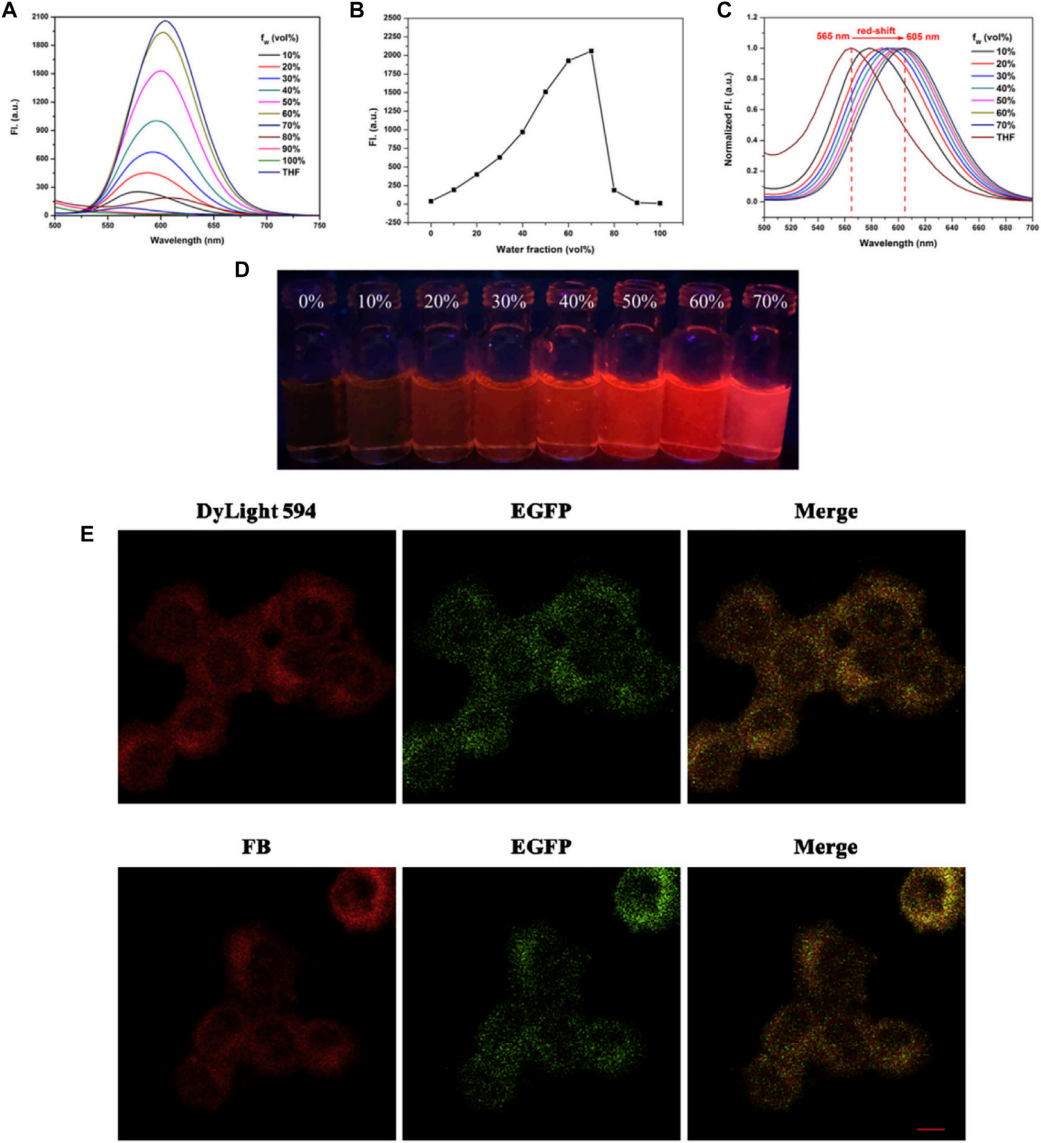
AIE characteristic study of the probe FB. **(A)** Fluorescence spectrum of the probe FB in THF-H_2_O solution. **(B)** Fluorescence intensity of the probe FB at 605 nm in various water fractions. **(C)** Normalized fluorescence spectrum of the probe FB in THF-H_2_O solution. **(D)** Photographs of the probe FB in THF-H_2_O solution under UV lamp (365 nm). **(E)** Cell staining images in the transfected PC-12 cells (Wang Y. et al., 2020).

Among the molecular, organelle, and cellular level, organelles may represent suitable targets for the detection, imaging, and treatment of diseases. AIEgens modified with various targeting groups could serve as superior imaging agents for monitoring of the dynamics of various biological process-related organelles and enzymes (Gu et al., 2017). Receptor targeting is one of the general strategies for cell imaging due to the overexpression of specific receptors, which is strongly linked to the progression of disease (Ko et al., 2019). Abnormal intracellular lipid droplets (LDs) are important biomarkers of multifarious diseases and are associated with cancer, obesity, fatty liver, Alzheimer’s disease, and other diseases. Thus, the visual monitoring of LDs is of great significance. Yuxuan Wang et al. (2020) combined hydrophobic AIEgen tissue polypeptide antigen (TPA) with lecithins via the condensation of Schiff base for targeted aggregation on LDs in HeLa cells with high SNR. Induced by UV light (493 nm), the real-time tracing of LDs was achieved to explain cellular processes (Figure 14).

**FIGURE 15:**
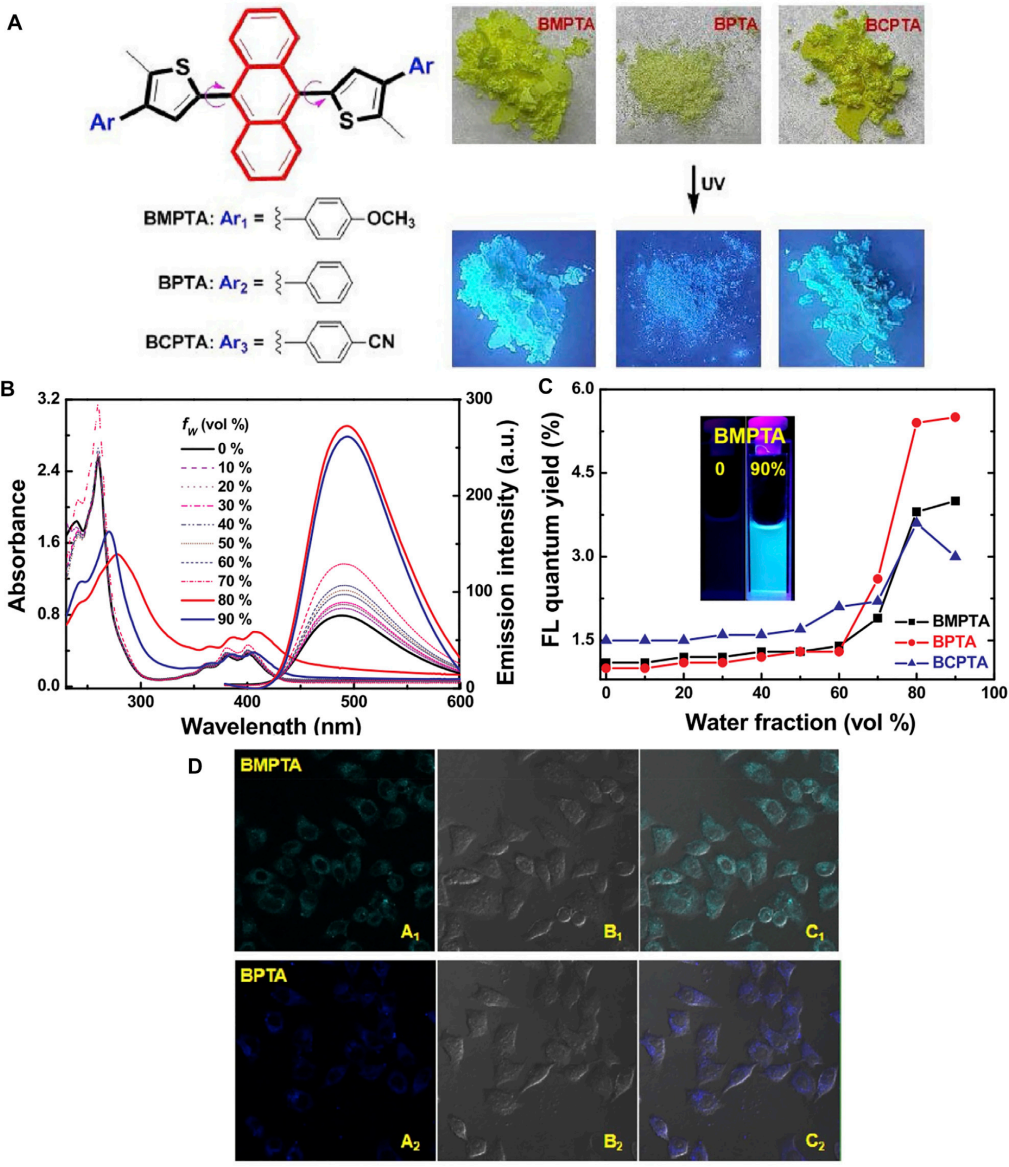
**(A)** Molecular structures of DTA derivatives and their photographic images under 365 nm UV light; **(B)** the absorption and fluorescence spectra of BMPTA in THF/H_2_O with different fractions; **(C)** their changes in absolute fluorescence quantum yield versus water fraction in THF; and **(D)** fluorescent confocal microscopy images of HeLa cells stained with 20 μM of BTA compounds for 30 min dark field images, bright field images, overlaid of dark and bright field images (Wang R. et al., 2020).

In addition, 9,10-dithienylanthracene (DTA) derivatives with a rigid plane structure often exhibit desirable AIE behaviors and fluorescence efficiency in the near-infrared region. Wang developed a novel fluorescence tracer with a similar structure (Wang R. et al., 2020). Multiple hydrogen bond interactions between neighboring molecules limit their intramolecular rotation, allowing for significant fluorescence enhancement (Figure 15). The results confirmed that substituent groups had a significant effect on their molecular packing, morphology observation, and optical properties. Moreover, the DTA molecules reported could be used fluorochrome in the nucleolus and localized uniformly in HeLa cells.

**FIGURE 16:**
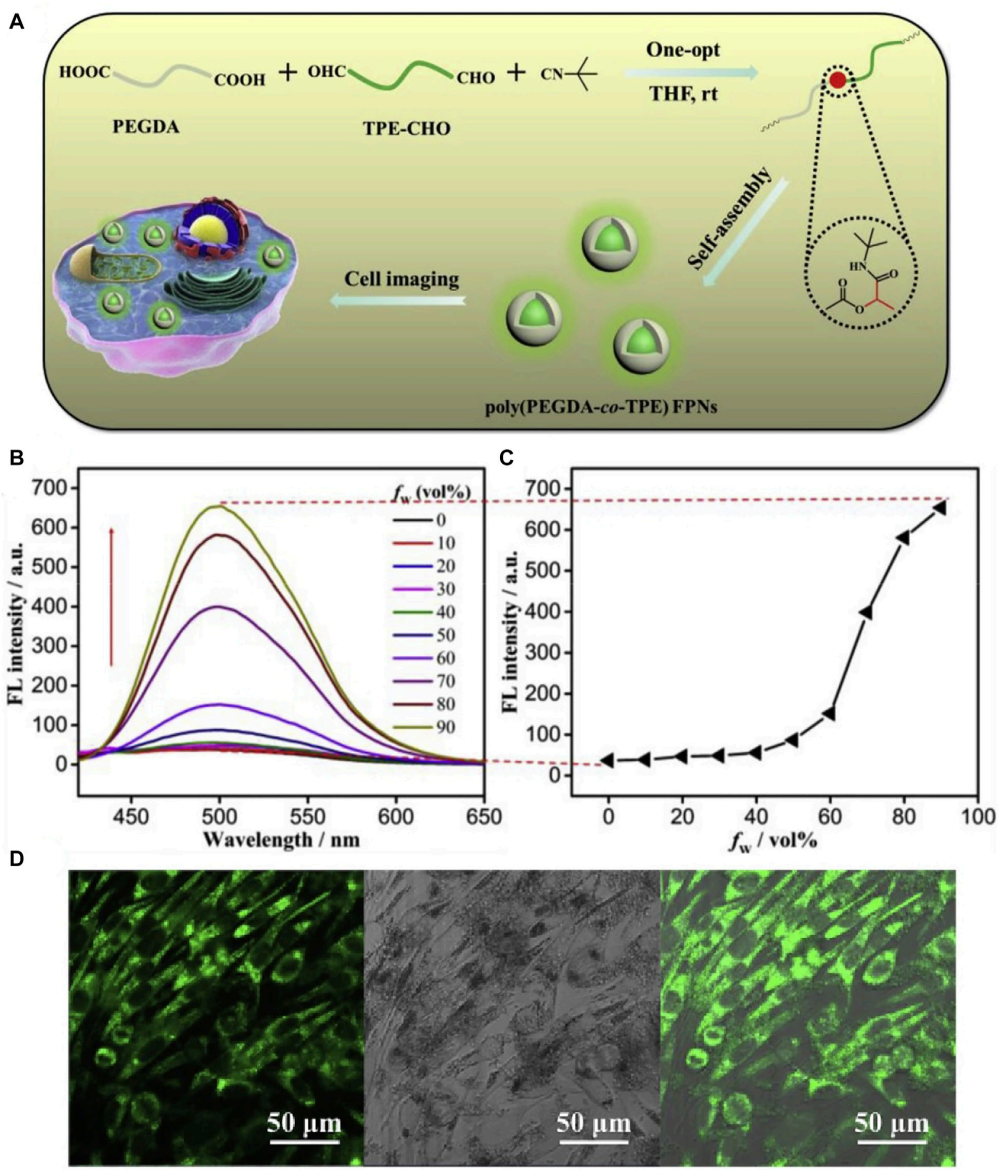
**(A)** Schematic showing the preparation of poly (PEGDA-co-TPE) FPNs for cell imaging; **(B)** the fluorescent spectra of poly (PEGDA-co-TPE) in DMF/water mixtures with different water fractions; **(C)** the relationship of fluorescent spectra of poly (PEGDA-co-TPE) versus water fraction in the mixed solution; and **(D)** CLSM images of L929 cells incubated with 40 μg ml^−1^ of poly (PEGDAco-TPE) FPNs (Jiang R. et al., 2020b).

Amphipathic poly (PEGDA-co-TPE) copolymers were synthesized *via* a catalyst-free one-pot Passerini reaction (Jiang R. et al., 2020b) under fairly mild experimental conditions. In comparison with small fluorescence molecules, the macromolecular colorants have high luminous efficiency, large Stokes shift, and remarkable water dispersibility (with the hydrophilic group), demonstrating the great potential for mapping and tracing target cells *in vivo* (Figure 16).

### Tissue imaging

Unlike cell imaging, tissue imaging by AIEgen offers two main benefits. The high luminescence efficiency and structural stability of AIE materials warranted improved *in vitro* tissue imaging; furthermore, with the assistance of fluorescence sensors, medical staff could define boundaries of pathological tissue and delineate tiny tumor nodules more precisely, remarkably improving the success rate of surgeries. As tumor resection surgery remains the preferred and most commonly used strategy, accurate intraoperative tumor resection is guided by optical imaging to address these severe problems taking advantage of its excellent sensitivity, real-time modality, super temporal resolution, and fine biosafety (Chen C. et al., 2020). This strategy was supported by the mapping performance of AIE probes, as a precise and effective means of tracing and treating pathological tissues.

**FIGURE 17:**
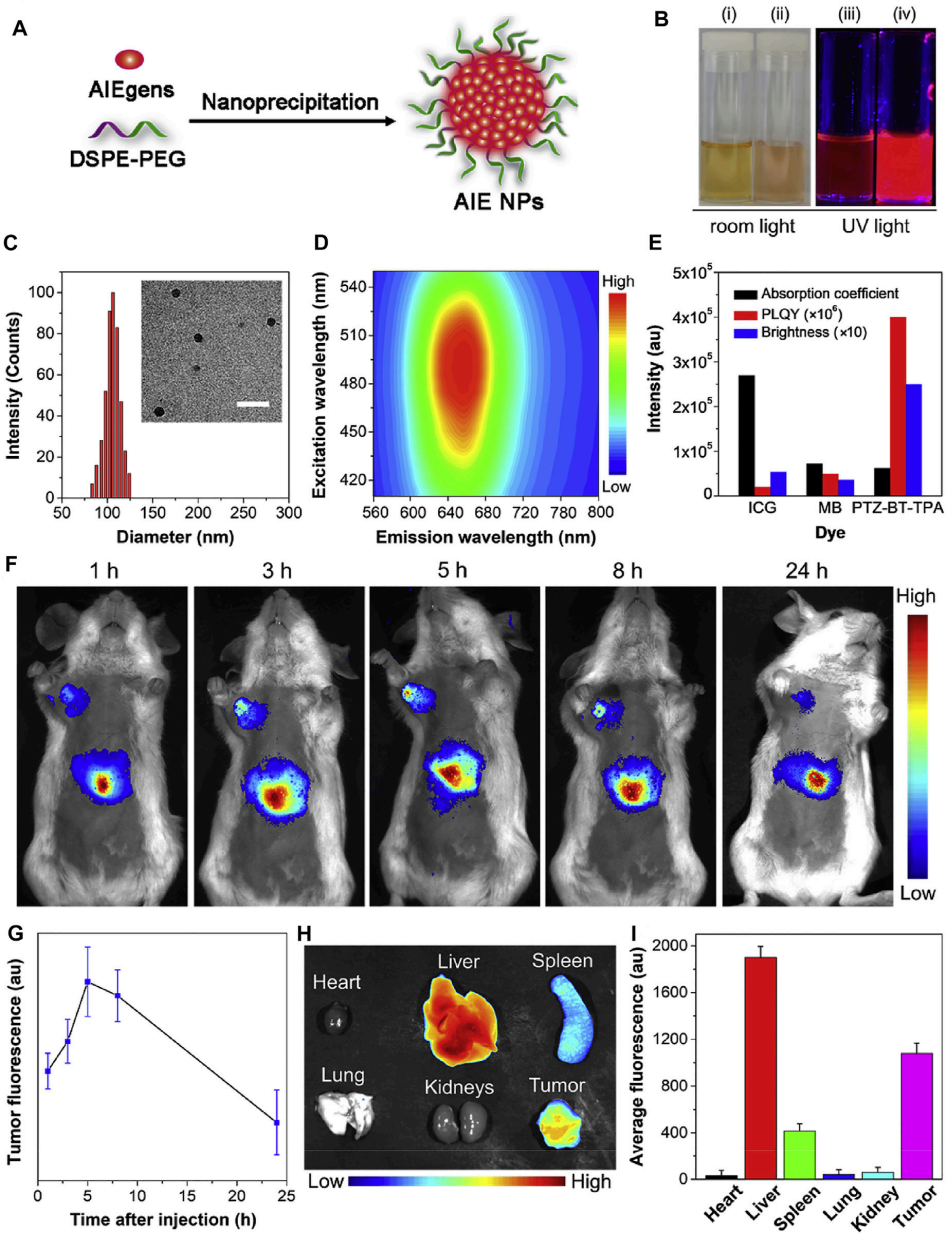
**(A)** Schematic illustration of the preparation of AIE-NPs through a nanoprecipitation method. **(B)** Photographs of PTZ-BT-TPA (i, iii) in THF and (ii, iv) as NPs under room and UV light (365 nm), respectively. **(C)** Representative DLS profile and TEM image of the AIE-NPs. Scale bar = 300 nm. **(D)** Photoluminescence excitation mapping of the AIE-NPs. **(E)** Comparison of the absorption coefficient, PLQY and brightness of ICG, MB and PTZ-BT-TPA in water. **(F)** In vivo fluorescence imaging of tumor-bearing mice and **(G)** the corresponding fluorescence intensity of tumor site at different time intervals after intravenous injection of the AIE-NPs. Data are presented as the means ± SD (*n* = 3). **(H)** Ex vivo fluorescence image and **(I)** fluorescence intensity of main organs (heart, liver, spleen, lung, and kidneys) and tumor after intravenous administration of AIE-NPs for 24 h.

Symmetric structure sometimes allows for the precise localization of tumor tissues. Qi et al. (2020) designed a novel dragonfly-shaped NIR molecule with the AIE structure of TPE (Figure 17). Through self-assembly, the nanoparticles possess exciting long-term tumor mapping capacity; the accumulation of different organs was also investigated, indicating that this molecule ensures the efficiency of *in vivo* tumor imaging.

**FIGURE 18:**
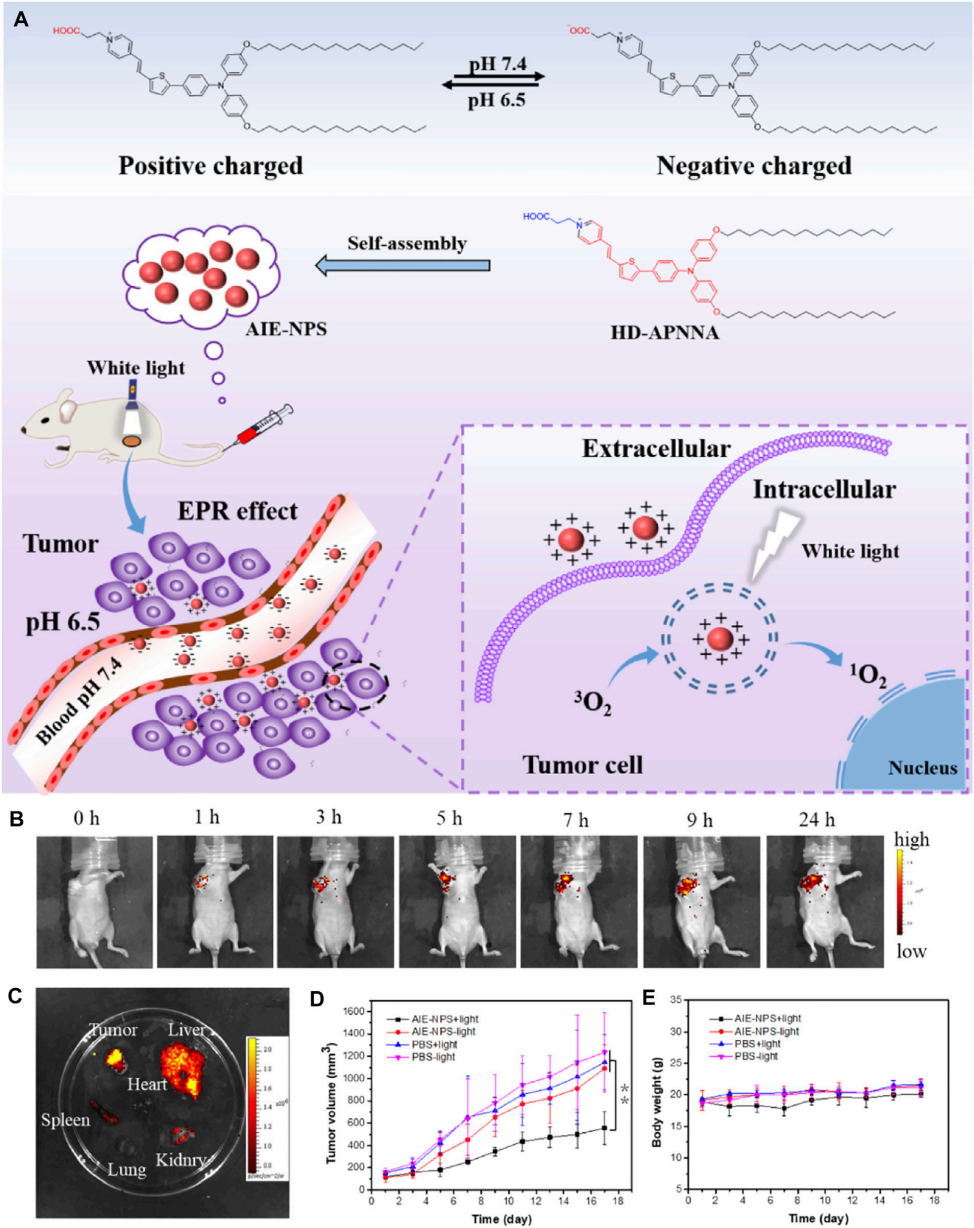
**(A)** Illumination of carrier-free AIE-NPs for tumor photodynamic therapy. **(B)** *In vivo* fluorescence images of HeLa tumor-bearing mice after intravenous injection with the AIE-NPs (1 mg/ml, 50 μL per mouse). **(C)** *Ex vivo* fluorescence images of organs and tumor at 9 h after intravenous injection of the AIE-NPs. **(D)** Volume growth curves of tumors at different time points posttreatment in different groups (n = 3, *p < 0.05, **p < 0.01. **(E)** Body weight measurement of the mice in each group (Cheng et al., 2020).

AIE materials are more than excellent tool of detection and exhibition. In recent years, aggregation-induced emission photosensitizers (AIE-PSs) with strong photosensitization perform well in the efficient tumor photodynamic therapy. For the design of functional materials, load factor is regarded as an important parameter of graft, modification, and composition. Cheng et al. (2020) designed novel pH-responsive nanoparticles self-assembled by amphiphilic AIE molecule with a 100% loading efficiency. As shown in Figure 18, the fluorescence quantum yield of AIE-NPs reached 56.7%, better than the probes commonly used in clinic. As a result, the AIE-NPs exhibited an effect on extracorporeal photodynamic therapy *in vitro* under white light (50 mW cm^−2^). The experiment using tumor-bearing mice verified the high tumor accumulation, penetration, and therapeutic efficiency of AIE-NPs.

AIE molecules can monitor key factors during biological processes with high precision and satisfying reliability in real time in addition to acting as the delivery platform of drugs, similar to the image-guided therapeutic response. The therapeutic AIE-active materials are still in the earlier developmental stages but have the potential to create a new direction toward the study of bio-optical material, and much more work should be accomplished in this promising pursuit.

## Conclusion and perspectives

In summary, we have detailed the recent research strategies of AIE molecules, which can potentially be used in biological testing and imaging applications. The recent synthesis of numerous AIE molecules has offered a great opportunity to study the photophysical properties of such compounds revealing the subtle mechanisms that led to this phenomenon.

Although tremendous development in the study of AIE molecules has been achieved, there are still plenty of opportunities and challenges remaining in this field. Achieving the following research goals will deepen our comprehension and help to facilitate the creation of innovative light-based biotechnologies: 1) broadening the scope of mechanisms for building novel AIE-based structures; 2) designing novel molecules with high light penetrability and high quantum yield; 3) combining of sensing and imaging strategies for the detection of target biomolecules and *in vivo* fluorescence imaging; and 4) designing and manufacturing of AIE biosensors with multi-model sensors and imaging capabilities (MRI, THz, photoacoustic imaging, etc.).

The development of novel AIE-based molecules/materials is highly desirable for many applications, e.g., biomolecular sensing, biological imaging, chemical sensing, as stimuli-responsive materials, and optoelectronic systems. The simplicity and promising advantages of AIE systems will encourage scientists to study new AIE materials, not only increasing the variety of molecules developed but also working toward biological and physiological applications for the accurate diagnosis and treatment of diseases.

**TABLE 1:** AIE-based fluorescent chemosensor for detecting of analytes.

Probe	AIE luminogens	Analyte	References
Terpyridine-based quaternary ammonium salt	terpyridine	Hg^2+^	Zhou et al. (2020)
TPE-based quaternary ammonium salt	TPE	Ce^4+^/ascorbic acid	Xiangqian Li et al. (2020)
Aliphatic amide salt	None	Cu^2+^	Qinghui Wang et al. (2020)
Aqueous light-harvesting systems	Oxalyldihydrazone	Cu^2+^	Guan et al. (2020)
Pyrrole-quinazoline derivative	Salicylaldehyde hydrazones	Cu^2+^/pyrophosphate	Wan-Wan Wang et al. (2020)
Bile acid derivative	Terpyridine	Zn^2+^/Cd^2+^	Chao Liu et al. (2020)
Self-assemblies of h4tcbpe-MA	TPE derivative	Melamine	Zejun Xu et al. (2020)
TPE derivative	TPE derivative	PA	Cihang Yan et al. (2020)
Dimethylamino Schiff base	AIE luminogens intramolecular hydrogen bonds	Methanol	Ruixue Zhao et al. (2020)
TPE-br	TPE	Sodium dodecyl sulfate	Feng et al. (2020)
Ferrocene conjugated quinoxaline derivatives	Quinoxaline derivatives	PA	Rajalakshmi and Palanisami (2020)

**TABLE 2:** AIE-based fluorescent probe for biosensing.

Probe	AIE luminogens	Target	References
Conjugate of anthraquinone and tetraphenylethylene	TPE	Cytomembrane	Lina Zhang et al. (2020b)
Mesoporous silica nanomaterials	TPA derivative	Cytoplasm	He et al. (2020)
cRGD-functionalized AIE dots	TPE	Groove of double-stranded DNA	Wang et al. (2015)
TPE-Py-NCS	TPE	Mitochondrion/lysosome	Gu et al. (2017)
Diaminomaleonitrile Schiff-base	Cyano-substituted stilbenes	ATP	Zhonglong Wang et al. (2020d)
LPS specific binding peptide	TPE	Lipopolysaccharide	Tang et al. (2020)
Rhodamine-phenothiazine	Phenothiazine derivative	Mitochondria	Jiande Dong et al. (2020)
Biosensors based on GO	Siloles	Bovine serum albumin (BSA)	Xu et al. (2012)
